# Bioinformatics analysis of the biological changes involved in the osteogenic differentiation of human mesenchymal stem cells

**DOI:** 10.1111/jcmm.15429

**Published:** 2020-05-28

**Authors:** Tingyu Fan, Rongmei Qu, Qinghe Yu, Bing Sun, Xin Jiang, Yuchao Yang, Xiaolan Huang, Zhitao Zhou, Jun Ouyang, Shizhen Zhong, Jingxing Dai

**Affiliations:** ^1^ Guangdong Provincial Key Laboratory of Medical Biomechanics and Department of Anatomy School of Basic Medical Science Southern Medical University Guangzhou China; ^2^ Zhujiang Hospital Southern Medical University Guangzhou China; ^3^ Central Laboratory Southern Medical University Guangzhou China

**Keywords:** bioinformatics analysis, human bone marrow mesenchymal stem cells (hBMSCs), osteogenic differentiation, microtubule

## Abstract

The mechanisms underlying the osteogenic differentiation of human bone marrow mesenchymal stem cells (hBMSCs) remain unclear. In the present study, we aimed to identify the key biological processes during osteogenic differentiation. To this end, we downloaded three microarray data sets from the Gene Expression Omnibus (GEO) database: GSE12266, GSE18043 and GSE37558. Differentially expressed genes (DEGs) were screened using the limma package, and enrichment analysis was performed. Protein‐protein interaction network (PPI) analysis and visualization analysis were performed with STRING and Cytoscape. A total of 240 DEGs were identified, including 147 up‐regulated genes and 93 down‐regulated genes. Functional enrichment and pathways of the present DEGs include extracellular matrix organization, ossification, cell division, spindle and microtubule. Functional enrichment analysis of 10 hub genes showed that these genes are mainly enriched in microtubule‐related biological changes, that is sister chromatid segregation, microtubule cytoskeleton organization involved in mitosis, and spindle microtubule. Moreover, immunofluorescence and Western blotting revealed dramatic quantitative and morphological changes in the microtubules during the osteogenic differentiation of human adipose‐derived stem cells. In summary, the present results provide novel insights into the microtubule‐ and cytoskeleton‐related biological process changes, identifying candidates for the further study of osteogenic differentiation of the mesenchymal stem cells.

## 1 INTRODUCTION

Bone defects caused by bone trauma, tumours and infection are common and refractory diseases encountered in orthopaedic clinical settings. The reconstruction of bone defects is challenging. Currently, the most common treatment strategy is repair by bone transplantation; however, limitations of the donor restrict its application, thereby affecting patient outcomes. Although allogeneic bone transplantation does not suffer limitations arising from a lack of suitable donors, this strategy is associated with antigenicity, which often results in transplant failure due to severe immune rejection.^1^, ^2^ In this context, bone tissue engineering is an indispensable strategy because it offers clinicians the choice of a variety of artificial bone substitutes made of metal, ceramic or polymer, thus providing great convenience for clinical treatment (eg reducing the transplant failure risk).^3^, ^4^ Bone tissue engineering requires three elements: seed cells, scaffold materials and bone inducers. Mesenchymal stem cells (MSCs) such as human bone MSCs (hBMSCs) and human adipose‐derived stem cells (hASCs) exhibit the potential for self‐proliferation and differentiation into adipogenic, osteogenic, chondrogenic and other lineages. Both cell types represent ideal seed cells for bone tissue engineering.^5^, ^6^ The osteogenic differentiation of stem cells is the basis for bone formation. Elucidation of the mechanisms underlying the osteogenic differentiation of seed cells should enable the development of techniques aimed at the osteogenic regulation of seed cells, with potential application in the construction of biomaterials for application in bone tissue engineering.^3^, ^6^

Both hBMSCs and hASCs are commonly used as seed cells in bone tissue engineering applications. Increasing evidence shows that the osteogenic differentiation of MSCs involves a series of signalling pathways^7^, ^8^, ^9^ such as BMP‐SMAD,^10^, ^11^ WNT/Catenin,^12^ Notch^13^, ^14^ and MAPK,,^15^ and complex regulatory networks formed by interactions between these pathways.^16^, ^17^ Transcription factors, such as TWIST and MSX2, may also be involved in the regulation of osteogenic differentiation.^18^, ^19^ In addition, certain physical stimuli promote the osteogenic differentiation of stem cells; for example, Heydari demonstrated that substrate stiffness and exposure to electromagnetic fields enhance the osteogenic potential of stem cells in the absence of chemical stimulation.^20^, ^21^, ^22^ Therefore, elucidation of the precise molecular mechanisms underlying osteogenesis is crucial to the development of bone tissue engineering applications and treatment strategies for bone defects.

Microarray techniques and bioinformatics analysis have been widely used to screen for genome‐level differences involved in osteogenic differentiation, enabling the identification of differentially expressed genes (DEGs) and functional pathways associated with osteogenic differentiation of stem cells.^23^, ^24^, ^25^, ^26^ However, the false‐positive rates of independent microarray analysis data make it difficult to obtain reliable results. In this study, three mRNA microarray data sets were downloaded from Gene Expression Omnibus (GEO) for analysis to identify DEGs between the control group and the induction group. We then carried out gene ontology (GO), Kyoto genome and genome encyclopaedia (KEGG) pathway enrichment analysis and protein‐protein interaction (PPI) network analysis to elucidate the underlying molecular mechanisms. In summary, a total of 240 DEGs, 10 hub genes and one important biological process (microtubule‐related) were identified, and the changes in microtubules may be a key factor in osteogenic differentiation of mesenchymal stem cells.

## 2 MATERIALS AND METHODS

### 2.1 Microarray data

Gene Expression Omnibus (http://www.ncbi.nlm.nih.gov/geo) is an international, public functional genomic database that collects high‐throughput resources, including gene expression data, ChIP‐seq and microarrays. GSE12266,^27^ GSE18043^28^ and GSE37558^29^ were downloaded from GEO. We obtained osteogenically induced hBMSC samples in early‐stage from these three data sets. The GSE12266 data set contained 4 uninduced samples (GSM308067, GSM308071, GSM308075 and GSM308079) and 4 osteogenic induction samples (GSM308069, GSM308073, GSM308077, GSM308081). GSE18043 contained three uninduced samples (GSM250019, GSM250020 and GSM250021) and three osteogenic induction samples (GSM451159, GSM451160 and GSM451161). GSE37558 contained four uninduced samples (GSM921574, GSM921575, GSM921576, GSM921577) and three osteogenic induction samples (GSM921581, GSM921582, GSM921583). Based on the platform annotation information, probes were transformed into corresponding gene symbols.

### 2.2 Identification of DEGs

We combined samples from the control group and the induction group in three data sets, and removed or averaged the probe sets without corresponding gene symbols or the genes with multiple probe sets, respectively. Then, the limma package was used to remove batch effect and identify DEGs. LogFC > 0.584963 (ie FC > 1.5) and *P*‐value < .05 were considered to indicate statistical significance.

### 2.3 KEGG and GO enrichment analyses of DEGs

Metascape (https://metascape.org/gp/index.html#/main/step1)^30^ is an analytical website that combines functional enrichment, interactome analysis, gene annotation and membership search to leverage over 40 independent knowledgebases within one integrated portal. KEGG is a database resource for elucidating high‐level functions and effects of the biological system.^31^, ^32^ Gene Ontology (GO) is a major bioinformatics initiative that for high‐quality functional gene annotation and analysing gene biological processes (BP), molecular functions (MF) and cellular components (CC).^33^ Metascape was used for analysing the function of DEGs. Min overlap = 3 and Min Enrichment = 1.5 were the screening conditions. *P* < .01 was considered statistically significant.

### 2.4 PPI network construction and module analysis

The PPI network was analysed using the Search Tool for the Retrieval of Interacting Genes (STRING; http://string‐db.org). Analysis of functional interactions between proteins was performed in order to elucidate the mechanisms of osteogenesis and development. An interaction with a combined score > 0.4 was selected and used to construct a PPI network with Cytoscape software. Cytoscape (version 3.7.1) is an open source bioinformatics software platform for visualizing molecular interaction networks.^34^, ^35^ Dense connected regions were analysed using Cytoscape's plug‐in molecular complex detection (MCODE). Our selection criteria were as follows: MCODE scores > 5, degree cut‐off = 2, node score cut‐off = 0.2, Max depth = 100 and k‐score = 2. KEGG and GO analyses were then performed using Metascape.

### 2.5 Selection and analysis of hub genes

The top 10 genes were obtained by MCC algorithm with Cytoscape's plug‐in cytoHubba. Protein expression profiles of hub genes at tissue level (low, medium, high) and gene expression level (normalized expression, NX) were obtained from the HPA (Human Protein Atlas) database. The gene expression scores of hub genes at the tissue level were obtained from the Bgee database (https://bgee.org).

### 2.6 Cell culture and osteogenic differentiation

Aseptic human adipose tissue was obtained from the Plastic Surgery Department of Nanfang hospital, digested with 0.15% type I collagenase for 40 minutes, terminated with growth medium and then centrifuged at 800 rpm for 5 minutes. The cells were plated in a 10‐cm dish, incubated in a 37°C incubator with 5% CO_2_, and the medium was replaced 24 hours later. Cells cultured for 3‐6 passages were used for seeding a 6‐cm dish at a density of 8000 cells/cm^2^. with the medium was replaced with osteogenic differentiation medium when cell confluence was ~80%. The medium was changed every 2 days. The osteogenic effects were identified by ALP and alizarin red staining at day14 and day 21, respectively.

Growth medium (GM) comprised Dulbecco's modified Eagle's medium (DMEM) with 10% foetal bovine serum (FBS; Gibco; California; USA), 1% penicillin/streptomycin (Gibco) and 2 ng/mL FGF.

Osteogenic differentiation medium (OS) contained 10% FBS, 100 nmol/L dexamethasone, 37.5 mg/L ascorbic acid, 10 mmol/L‐glycerophosphate sodium, 10 nmol/L Vit D3 and 2 ng/mL FGF.

### 2.7 Immunofluorescence analysis

Cells were fixed with 4% paraformaldehyde for 10 minutes, and membranes were ruptured by 0.1% Triton X‐100 for 5 minutes. The samples were blocked with 2% BSA for 1 hour at room temperature, then incubated with α‐tubulin antibody (ab7291; Abcam), 4°C overnight. Incubation with the second antibody was performed at room temperature for 1 hour the next day. Nuclei were labelled with DAPI. Samples were sealed with glycerine gelatin. Images were collected using a confocal microscope (LSM 880 with Airyscan; Carl Zeiss) and analysed with software ZEN‐blue‐edition (Carl Zeiss).

### 2.8 Western blotting

Protein samples were extracted from cells using a protein extraction kit (KeyGEN Biotech), separated by sodium dodecyl sulphate‐polyacrylamide gel electrophoresis (SDS‐PAGE) and transferred to a polyvinylidene fluoride (PVDF) membrane (Millipore). The membranes were incubated with α‐tubulin antibody (ab7291; Abcam) after blocking with 5% milk, at 4°C, overnight. Incubation with the secondary antibody was performed at room temperature for 1 hour. FDbio‐Dura ECL kit (Fudebio) was used to detect the signal. The results of Western blotting (WB) were analysed with ImageJ software and plotted with GraphPad Prism 7.0 software.

### 2.9 Alizarin red staining and alkaline phosphatase staining

After fixation with 4% paraformaldehyde for 10 minutes, cells were incubated with fresh alizarin red solution (Cyagen) for 5 minutes. Images were obtained using a Olympus microscope.

After fixation with 4% paraformaldehyde for 10 minutes, cells were incubated with alkaline phosphatase (ALP) staining liquid (Beyotime Biotechnology; Shanghai; China) for 30 minutes. Images were obtained using Olympus microscope.

### 2.10 Statistical analyses

All experiments were independently repeated at least three times. Statistical analysis was performed with GraphPad Prism 7.0 software. One‐way analysis of variance (ANOVA) was used to identify significant differences, and *P* < .05 was considered to indicate statistical significance.

## 3 RESULTS

### 3.1 Identification of DEGs during hBMSC osteogenesis

**Figure 1:**
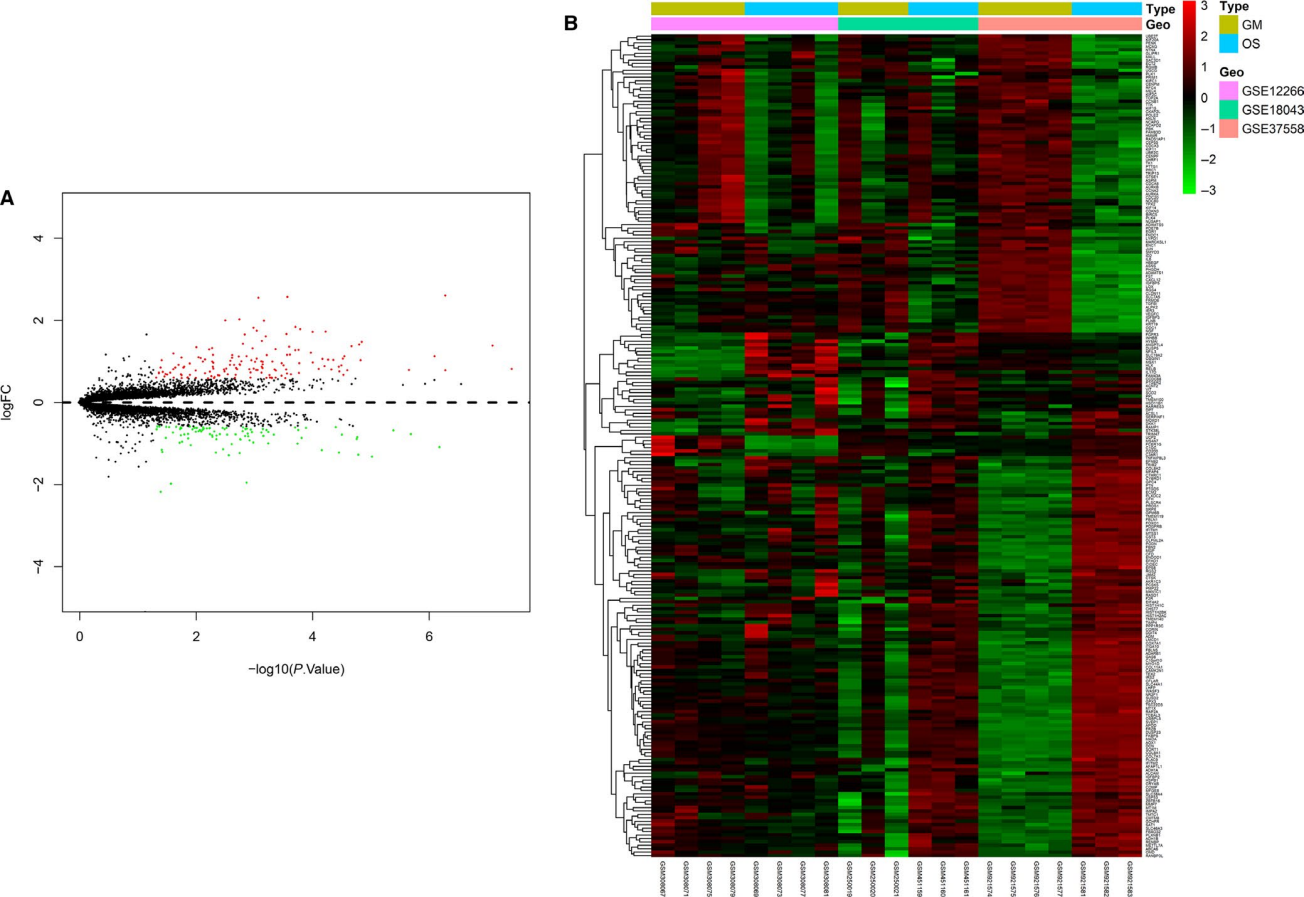
DEGs. A volcano plot (A) and heat map (B) showing the 240 differentially expressed genes. Red colour indicates up‐regulated genes, and green indicates down‐regulated genes. Group GM: cells treated with growth medium. Group OS: cells treated with osteogenic differentiation. *P* < .05, logFC > 0.584963

Because different osteogenic induction guidelines were used for the three data sets (GSE12266, GSE18043 and GSE37558 (Table S1)), we performed a preliminary comparison of the distribution of their DEGs prior to conducting formal experiments. The volcanic map and the circular network analysis map obtained with Metascape showed that the distribution of their DEGs is different (Figure [Link], [Link], [Link]A‐D). GSE12266 and GSE18043 were detected with the GPL570 platform, while GSE37558 was detected with the GPL6947 platform. This suggests that differences in detection platforms or induction schemes used may affect hBMSC osteogenic differentiation and induce different cellular responses. Therefore, in order to further understand the universality of the cell biological response of hBMSCs during osteogenic differentiation, we combined samples from the three data sets and analysed them. After normalizing the data, 240 DEGs were identified, included 147 up‐regulated genes and 93 down‐regulated genes, as shown in the volcano map and heat map (Figure 1A‐B).

### 3.2 KEGG and GO enrichment analyses of DEGs

**Figure 2:**
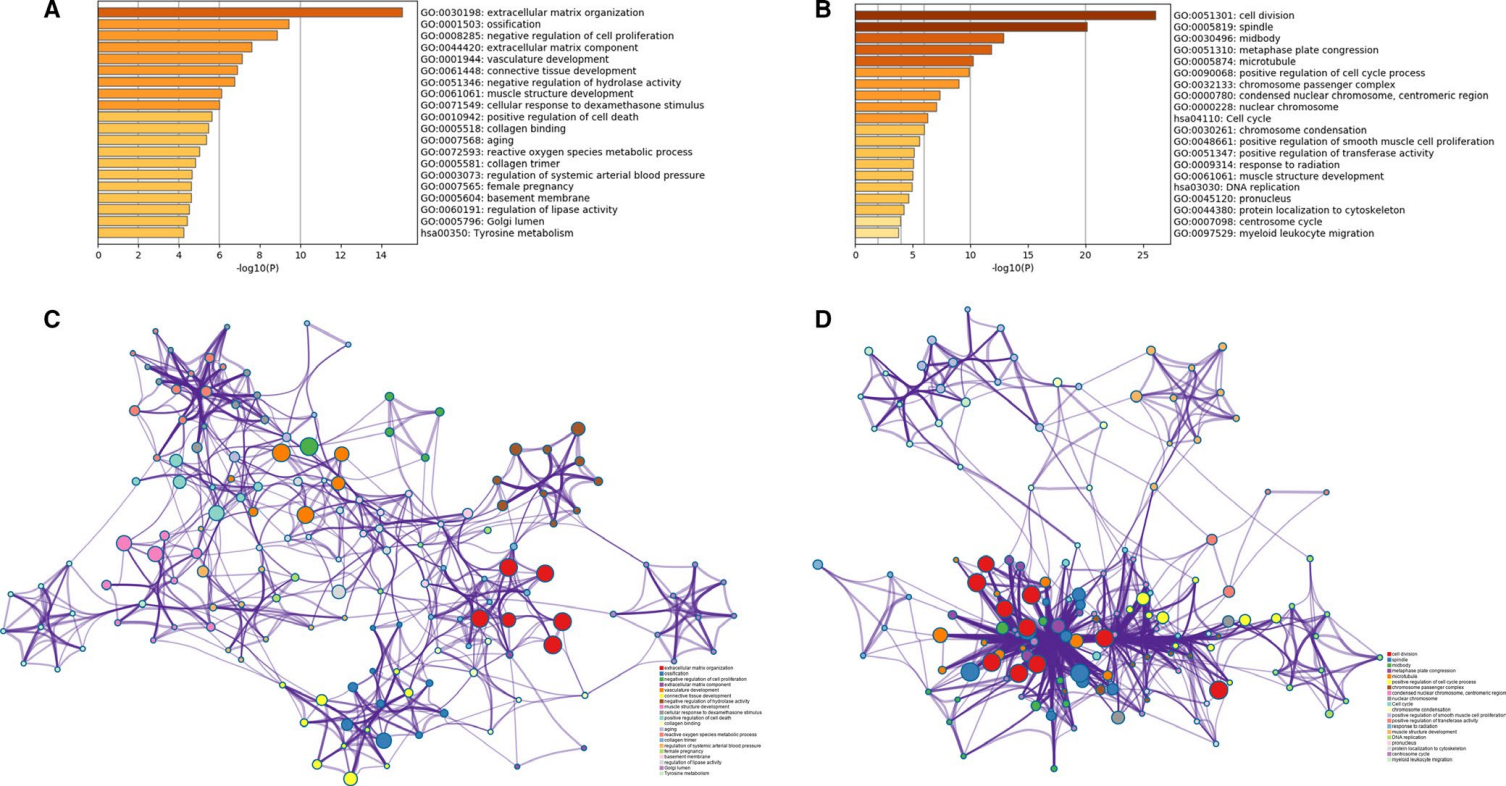
Functional enrichment analysis of DEGs Bar graph showing the top 20 results from enrichment analyses of up‐regulated genes (A) and down‐regulated genes (B). *P* value is shown in colour. The network of enriched terms of up‐regulated genes (C) and down‐regulated genes (D), showing the top 20. Each cluster ID is indicated with a specific colour

In order to analyse the biological classification of DEGs, we performed functional enrichment analysis of up‐regulated and down‐regulated genes, respectively. GO analysis results showed that the up‐regulated genes were mainly enriched in extracellular matrix organization, ossification, negative regulation of cell proliferation, vasculature development and positive regulation of cell death (Figure 2A and C), while the down‐regulated genes were significantly enriched in cell division, spindle, midbody, metaphase plate congression and microtubules (Figure 2B and D). KEGG pathway analysis indicates that the up‐regulated genes were mainly enriched in tyrosine metabolism, while the down‐regulated genes were mainly enriched in cell cycle DNA replication.

### 3.3 PPI network construction and module analysis

**Figure 3:**
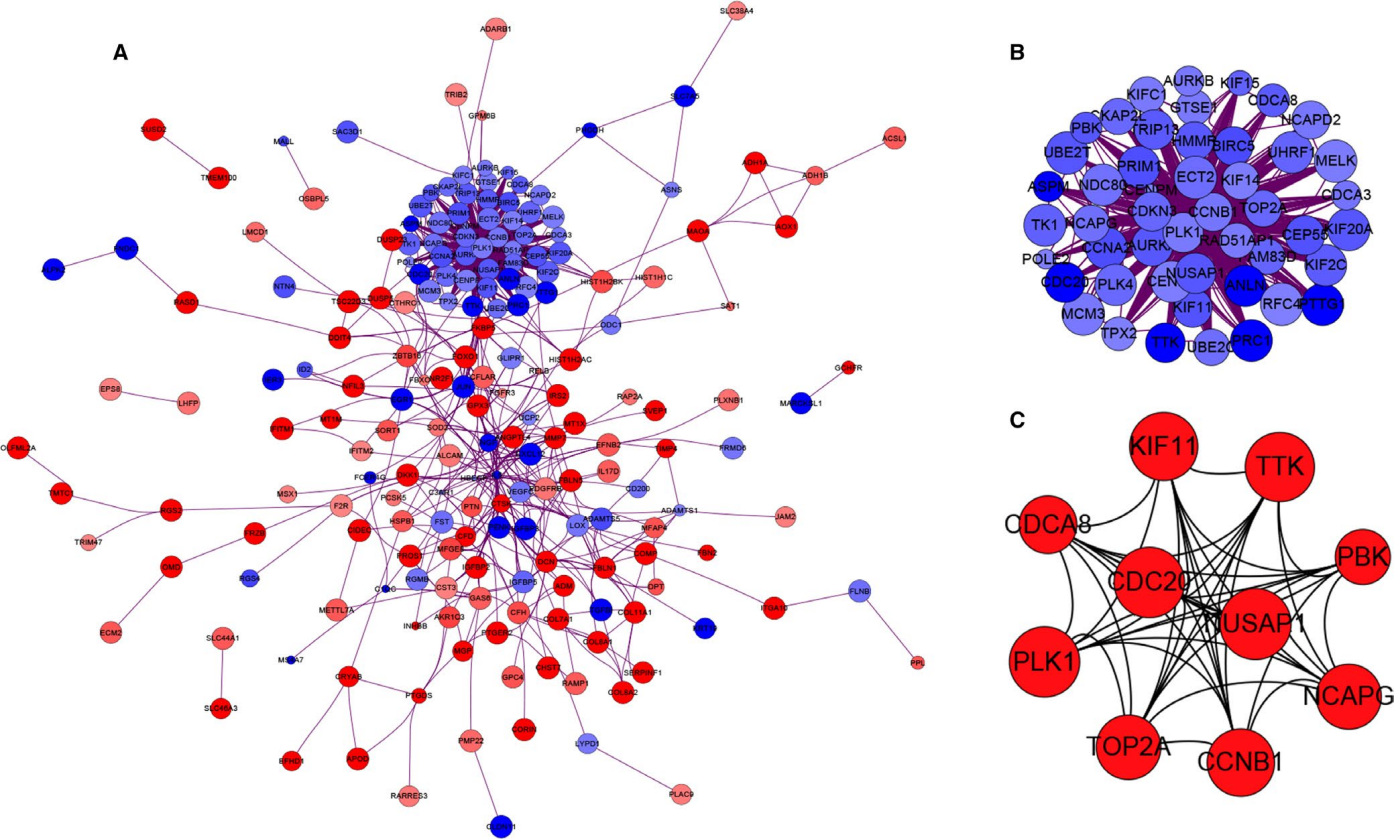
PPI network construction and module analysis (A) The PPI network of DEGs. The up‐regulated genes are marked in red, while the down‐regulated genes are marked in blue. The greater the difference in expression, the darker the colour. The size of nodes represents the difference in expression; the larger the size, the more significant the *P* value. B, The densest connected regions (48 nodes, 1056 edges) in the PPI network were identified with Cytoscape. C, Ten hub genes were identified in the densest connected regions with MCC algorithm, using cytoHubba. The score is indicated in red colour. Darker colour indicates a higher score

The PPI network of DEGs and most dense connected regions (48 nodes, 1056 edges) were obtained by Cytoscape (Figure 3A‐B). Functional enrichment analysis of the genes in this densest region showed that they were mainly enriched in cell division, spindles, cell cycle phase transition, midbody and microtubule‐related complexes (Figure [Link], [Link], [Link]A‐C).

### 3.4 Selection and analysis of hub genes

**Figure 4:**
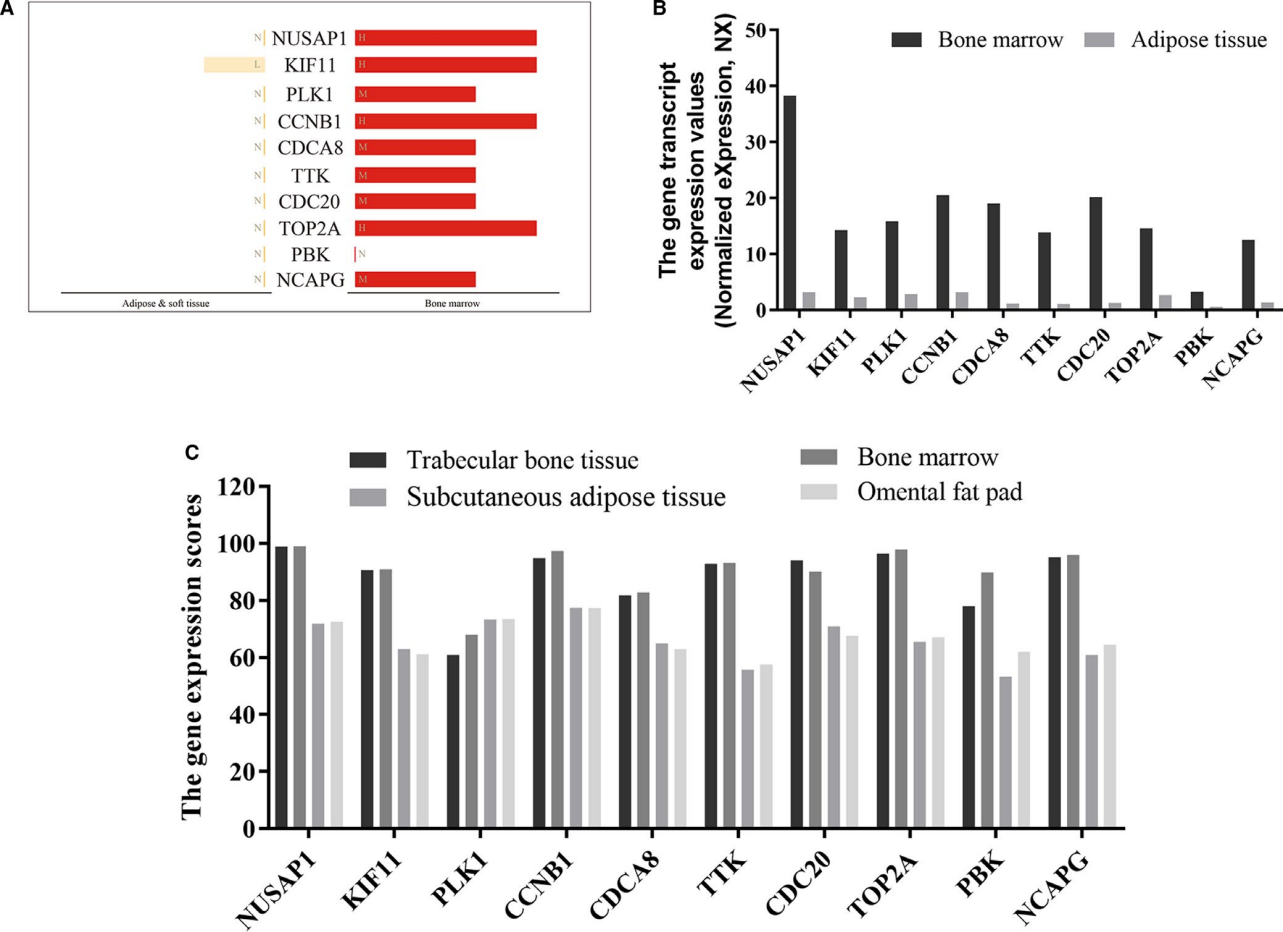
The expression analysis of hub genes in human tissues The analysis and summary of protein expression (A) and gene expression (B) of hub genes in human tissues from HPA database. H: high; M: medium; L: low; N: not detected. C, The gene expression scores of hub genes in human tissues were compared using the Bgee database. All scores were between 0 and 100. The maximum score was 100, and the minimum score was 0. A low score indicates that the gene was expressed at low levels in this tissue

Ten genes were identified as hub genes using the plug‐in cytoHubba in Cytoscape. The gene symbols, abbreviations and functions are shown in Table 1. According to the literature, osteogenic differentiation and adipogenic differentiation of stem cells tend to be the opposite of each other: stem cells are more likely to differentiate into adipogenic cells in an environment with lower substrate stiffness, and more likely to differentiate into osteogenic cells in an environment with greater substrate stiffness. Therefore, we compared the protein (Figure 4A) and gene expression levels (Figure 4B) of hub genes between human bone marrow and adipose tissue with the HPA database, and used this as a preliminary reference for identifying whether these genes were differentially expressed during osteogenesis. The results showed that, at the protein level, NUSAP1, KIF11, CCNB1 and TOP2A were highly expressed, while PBK was not detected, in bone marrow; in contrast, KIF11 was expressed at low levels, while expression of the other genes was not detected in adipose tissue (Figure 4A). The gene expression levels of these 10 hub genes in bone marrow were all higher than in adipose tissue (Figure 4B). Subsequently, we compared the gene expression scores of hub genes in trabeculae bone tissue, bone marrow, subcutaneous adipose tissue and the omental fat pad using data obtained from the Bgee database. Data showed that the gene expression scores of NUSAP1, KIF11, CCNB1, CDCA8, TTK, CDC20, TOP2A, PBK and NCAPG in trabecular bone tissue and bone marrow were higher than that in subcutaneous adipose tissue and the omental fat pad. *PLK1* was the only gene whose expression score was higher in the subcutaneous adipose tissue and omental fat pad than in trabecular bone tissue and bone marrow. Therefore, we believed that the expression of these 10 hub genes might differ between bone tissue and adipose tissue, and speculated that they may represent key genes in the process of osteogenic differentiation.

**Table 1:** Ten hub genes and their functions

Gene symbol	Description	Function
*NUSAP1*	nucleolar and spindle associated protein 1	Microtubule‐related proteins that promote the formation of mitotic spindles and microtubules
*KIF11*	kinesin family member 11	Assists in spindle formation during mitosis
*PLK1*	polo‐like kinase 1	Assists in spindle formation
*CCNB1*	cyclin B1	Required for the control of G2/M (mitosis) of the cell cycle
*CDCA8*	cell division cycle associated 8	Is essential for chromatin‐induced microtubule stabilization and spindle assembly
*TTK*	TTK protein kinase	Regulates cell proliferation; is crucial for the arrangement of chromosomes during mitosis and is necessary for centrosomal replication
*CDC20*	cell division cycle 20	Regulates the formation of synaptic vesicle clustering at active zone to the pre‐synaptic membrane in post‐mitotic neurons
*TOP2A*	DNA topoisomerase II α	Is crucial for the proper separation of chromosomes
*PBK*	PDZ binding kinase	The encoded protein may be involved in the activation of lymphocytes and support testicular functions
*NCAPG*	non‐SMC condensin I complex subunit G	Required for chromatin condensation

### 3.5 Microtubule changes during osteogenic differentiation of stem cells

**Figure 5:**
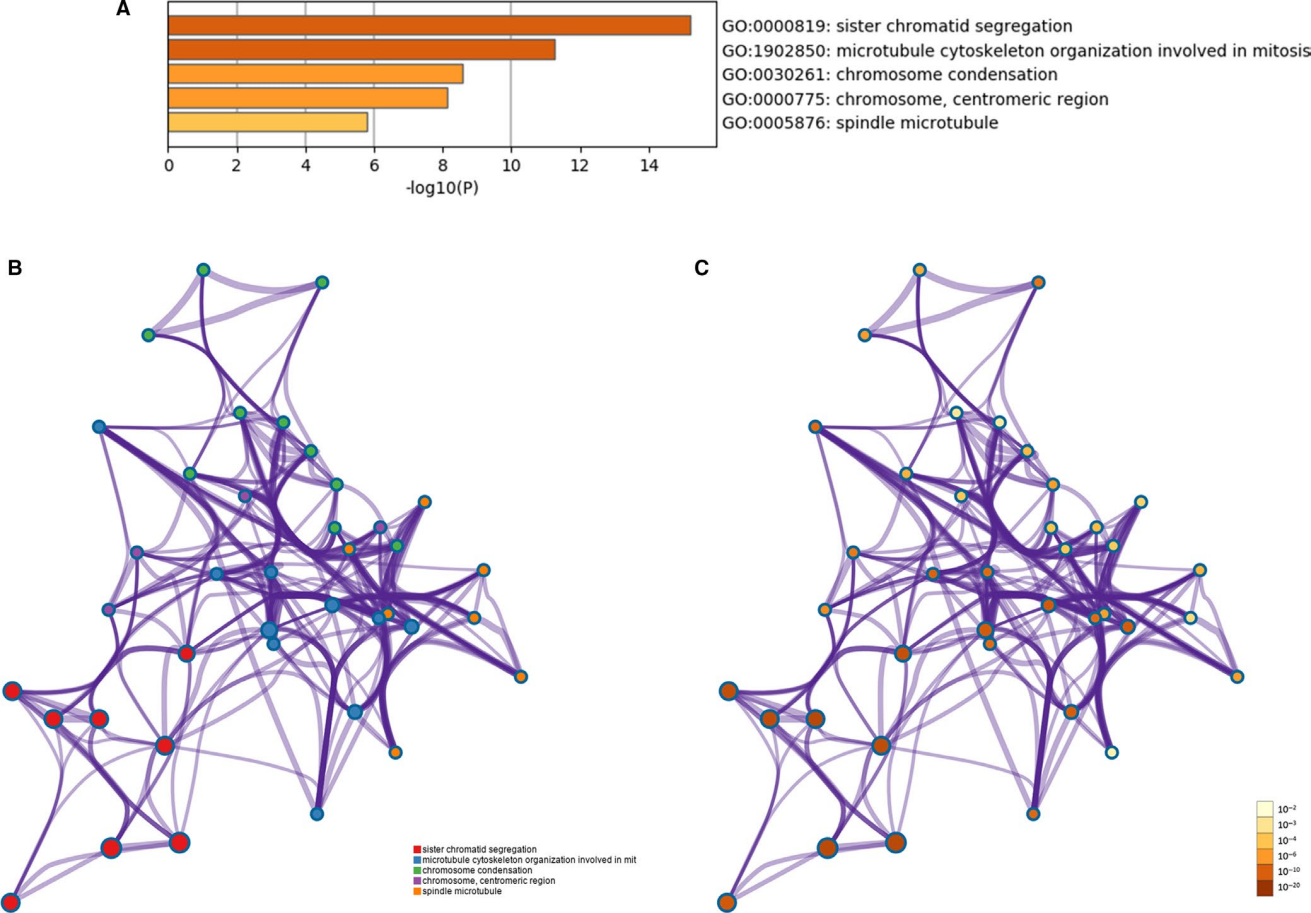
Functional enrichment analysis of hub genes (A) Bar graph of GO analyses of hub genes. *P* value is shown in colour. The network of enriched terms of hub genes; colours represent the same cluster ID (B) and *P*‐value (C)

Functional enrichment analysis showed that 10 hub genes were mainly concentrated in three biological processes (BP), namely sister chromatid segregation, microtubule cytoskeleton organization involved in mitosis, and chromosome condensation, as well as two cell components (CC) that is chromosome, centromeric region and spindle microtubule (Figure 5A‐C, Table 2).

**Table 2:** Functional enrichment analysis of hub genes

Term	Description	Count in gene set	‐LogP	Gene symbol
GO:0000819(BP)	Sister chromatid segregation	9	15.21400932	*CCNB1, CDC20, PLK1, TOP2A, TTK, NUSAP1, CDCA8, NCAPG, KIF11*
GO:1902850(BP)	Microtubule cytoskeleton organization involved in mitosis	9	11.27012818	*CCNB1, CDC20, KIF11, PLK1, TTK, NUSAP1, CDCA8, TOP2A, PBK*
GO:0030261(BP)	Chromosome condensation	6	8.581894105	*CCNB1, TOP2A, NUSAP1, NCAPG, PLK1, KIF11*
GO:0000775(CC)	Chromosome, centromeric region	6	8.122475997	*CCNB1, PLK1, TTK, CDCA8, NCAPG, NUSAP1*
GO:0005876(CC)	Spindle microtubule	4	5.784920985	*KIF11, PLK1, NUSAP1, CDC20*

Abbreviations: BP, biological processes; CC, cellular components; GO, Gene Ontology.

**Figure 6:**
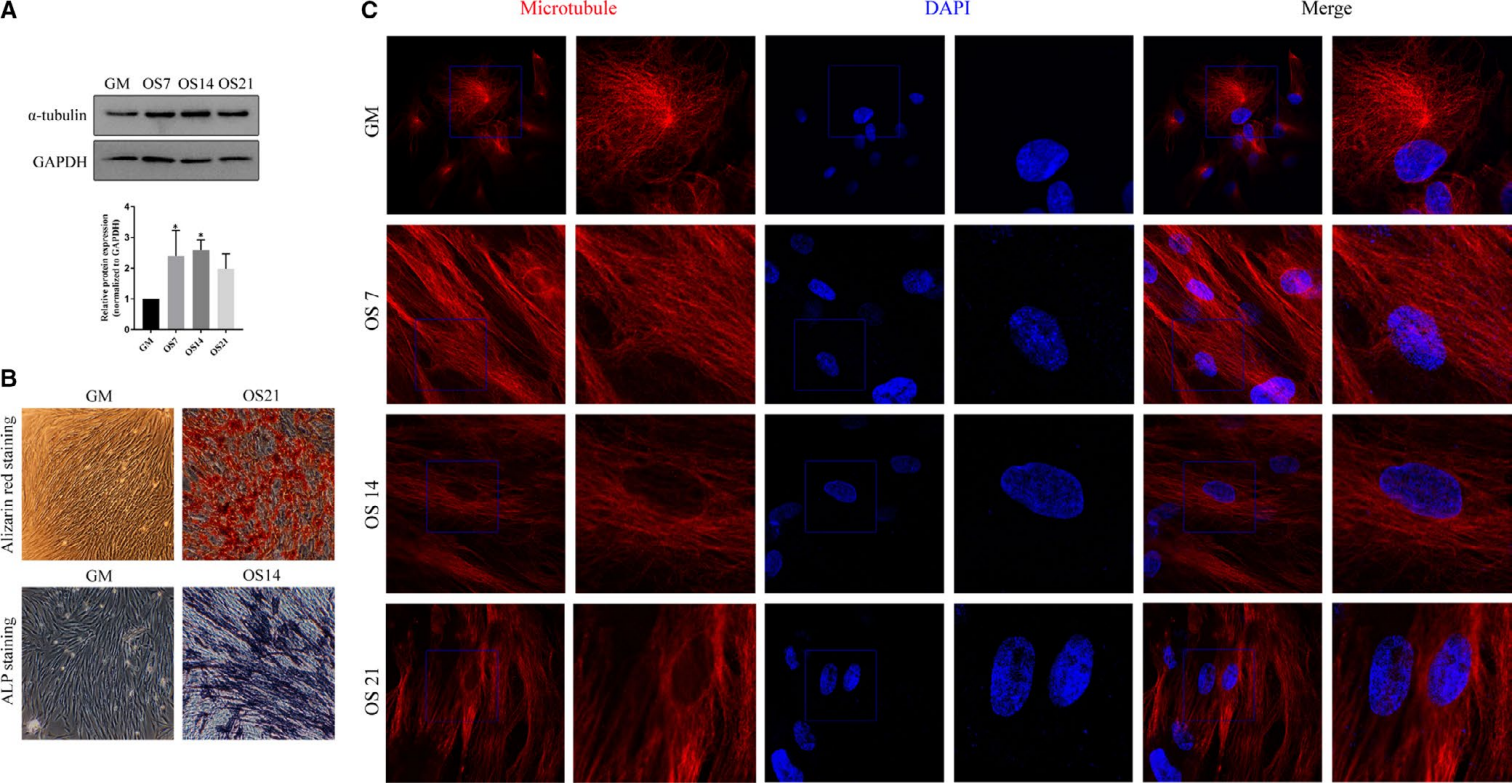
Microtubule changes during osteogenic differentiation of hASCs (A) Protein expression analysis of α‐tubulin; the upper is the band diagram for the Western blot, and the lower is the corresponding statistical analysis diagram. *GAPDH* was used as the internal reference gene. The results are presented as Mean ± SD, n > 3. *compared with GM, *P* < .05. B, Alizarin red staining and ALP staining. Bar = 50 µm. C, Morphological changes in microtubules during the osteogenic differentiation of hASCs. Bar = 10µm. Microtubules are marked in red, the nucleus in blue. GM: cells cultured in growth medium; OS7, OS14 and OS21: cells cultured in osteogenic differentiation medium for 7, 14 and 21 d, respectively

However, microtubule activity is essential for each of these biological processes and cell components. Therefore, we speculated that microtubule dynamics play an important role during the osteogenic differentiation of hBMSCs. In order to verify the universality of microtubule changes during osteogenic differentiation of mesenchymal stem cells, we detected the changes in morphology and protein expression in microtubules during osteogenic differentiation of hASCs, through immunofluorescence and WB experiments, respectively. WB results showed that the protein expression of α‐tubulin increased during osteogenesis (Figure 6A). The immunofluorescence images showed that in the GM group, microtubule filaments were radially scattered in the cytoplasm; further, an area of highly aggregated microtubules was observed near the nucleus (pointed to by the arrow in the GM group). In accordance with previous findings, we speculated that this represented the centrosomes, which are composed of microtubules. In the OS 7 group, this highly aggregated area was not observed; instead, microtubule filaments were arranged in parallel along the cell axis. The same morphological changes in microtubules occurred in the OS14 and OS21 groups (Figure 6C). These results suggest that the microtubule‐related changes are a representative biological process that occurs during the osteogenic differentiation of stem cells.

## 4 DISCUSSION

Mesenchymal stem cells, including hBMSCs and hASCs, have been proved to be ideal seed cells for bone tissue engineering. These cells are often used to assess the biocompatibility of a scaffold and its effectiveness in inducing bone formation. Complete elucidation of the mechanism underlying the osteogenic differentiation of stem cells should enable researchers to design suitable bone filling materials for patients, with more efficacious induction of bone tissue regeneration. Microarray technology enables exploration of the overall picture of genetic changes in stem cell osteogenesis and has been proved to be a useful method for identifying novel biomarkers.

In this study, we collected and analysed samples of hBMSCs undergoing early osteogenic differentiation, from three mRNA microarray data sets. A total of 240 DEGs were screened in the three data sets, and 147 up‐regulated genes and 93 down‐regulated genes were identified. Enrichment analysis of GO and KEGG was carried out to explore the interaction between DEGs. The up‐regulated genes were mainly enriched in extracellular matrix organization, ossification, negative regulation of cell proliferation, vasculature development, positive regulation of cell death and tyrosine metabolism, while the down‐regulated genes were significantly enriched in cell division, spindle, midbody, metaphase plate congression, microtubules, cell cycle and DNA replication. Up‐regulated genes were obviously enriched in bone formation, confirming that osteogenic differentiation of hBMSCs could be induced in three different osteogenic differentiation media. It has been shown that optimized extracellular matrix (ECM) induces stronger osteogenic effects in mesenchymal stem cells.^36^, ^37^ Recent studies show that stem cells can mechanically sense the stiffness of their microenvironment, and that substrate stiffness affects their differentiation. Extracellular matrix stiffness is clearly transduced into gene expression by adhesion and cytoskeletal proteins to regulate cell fate, and ECM‐related cytoskeletal recombination directly affects cell fate.^38^, ^39^, ^40^ Our findings are consistent with these previous results.

Among these 10 hub genes, we found that the interactions between bone formation and hub genes KIF11, PLK1, CDCA8, TTK, CDC20 and NCAPG have not been reported. Using ArrayExpress data, Wuxun et al also predicted in 2019 that NUSAP1 and PBK are key regulatory genes in osteogenic differentiation of hBMSCs.^41^ However, the expression of *TOP2A* in osteogenesis is controversial. Some studies have suggested that *TOP2A* is expressed in osteoblasts, and that parathyroid hormone can suppress the proliferation of osteoblasts partly by targeting *TOP2A* expression.^42^ Yamagishi suggested that TOP2A plays a role in the formation of osteoclasts,^43^ while Feister reported that TOP2A is not expressed in mature osteoblasts on the surface of trabeculae.^44^ CCNB1 can regulate the proliferation of bone marrow stem cells^45^, ^46^; however, the relationship between CCNB1 and osteogenic differentiation remains poorly understood.

According to the HPA and Bgee online database, we found that the expression of these hub genes in bone marrow and trabeculae was different from that in soft tissues such as adipose tissue (Figure 4A‐C). GO analysis showed that these genes were highly related to sister chromatid segregation, microtubule cytoskeleton organization involved in mitosis, chromosome condensation and spindle microtubule. All of these biological processes involve microtubule activity; in particular, proliferation and chromosomal segregation are mediated by microtubule‐based mitotic spindles and about 200 essential microtubule‐related proteins.^47^, ^48^, ^49^, ^50^ Furthermore, centrosomes, which are formed by microtubules, are necessary for rapid and accurate chromosome segregation.^51^, ^52^, ^53^, ^54^

Mitosis results in the formation of two new cells with the same genetic information.^55^ This involves deep remodelling of the microtubule network, including kinetochore microtubules that connect to the kinetochore of chromosomes, interpolar and central spindle microtubules that locate the furrow during cell division, and astral microtubules that anchor the furrow to the cellular cortex.^56^, ^57^ Microtubules are the core cytoskeletal filaments with a series of important cellular functions in eukaryotic cells, acting as structural scaffolds, cellular highways, force generators and signal platforms.^58^ As a result, microtubule production is tightly regulated in cells.^59^ The biological processes corresponding to the present hub genes involve extensive spatial, temporal and dynamic regulation of microtubules.^58^, ^59^, ^60^ Therefore, microtubule‐related changes in the osteogenic differentiation of stem cells were the focus of the present study.

To verify the universality of microtubule‐related changes in the osteogenic differentiation of stem cells, we performed preliminary verification in hASCs. Western blotting results showed that microtubules showed significantly increased protein expression during osteogenesis. Immunofluorescence images indicated that microtubules underwent drastic morphological changes during osteogenic differentiation, from radial to parallel arrangement. Although more detailed analysis is necessary to confirm the observed changes in microtubule‐related cell division and spindle during osteogenic differentiation, the present findings indicate that microtubule‐related changes play a major role in the osteogenic differentiation of stem cells and are thus worthy of further exploration.

In summary, the purpose of this study is to identify DEGs that may be involved in the occurrence and progression of osteogenic differentiation of stem cells. A total of 240 DEGs and 10 hub genes were identified, as well as non‐negligible biological changes—microtubule‐related changes. These results provide the basis for further study of osteogenic differentiation of stem cells; in particular, further research should aim to clarify the biological functions of the hub genes in this context. In the future, we intend to determine which of the present hub genes are most closely related to microtubules in order to further explore the osteogenic differentiation of stem cells.

## CONFLICT OF INTEREST

The authors declare no conflict of interest.

## AUTHOR CONTRIBUTION

**Tingyu Fan:** Conceptualization (lead); Data curation (lead); Formal analysis (equal); Methodology (equal); Resources (equal); Software (equal); Writing‐original draft (lead); Writing‐review & editing (equal). **Rongmei Qu:** Conceptualization (lead); Data curation (lead); Formal analysis (equal); Methodology (equal); Resources (equal); Software (equal); Writing‐original draft (equal); Writing‐review & editing (equal). **Qinghe Yu:** Data curation (equal); Investigation (equal); Methodology (equal). **Bing Sun:** Data curation (equal); Formal analysis (equal); Investigation (equal). **Xin Jiang:** Data curation (equal); Formal analysis (equal); Investigation (equal). **Yuchao Yang:** Data curation (equal); Formal analysis (equal); Investigation (equal). **Xiaolan Huang:** Data curation (equal); Formal analysis (equal); Investigation (equal). **Zhitao Zhou:** Data curation (equal). **Jun Ouyang:** Conceptualization (lead); Project administration (supporting); Supervision (supporting); Writing‐original draft (equal); Writing‐review & editing (equal). **Shizhen Zhong:** Conceptualization (lead); Funding acquisition (supporting); Project administration (supporting); Supervision (supporting); Writing‐original draft (equal); Writing‐review & editing (equal). **Jingxing Dai:** Conceptualization (supporting); Investigation (supporting); Project administration (supporting); Supervision (supporting); Visualization (lead); Writing‐original draft (supporting); Writing‐review & editing (supporting). 

## ETHICAL APPROVAL

This study was approved by the Ethics Committee of the School of Basic Medical Sciences, Southern Medical University.

## Supporting information

Fig S1

Fig S2

Sup info

## Data Availability

All the supporting data can be downloaded.

## References

[1] Moradi SL , Golchin A , Hajishafieeha Z , et al. Bone tissue engineering: Adult stem cells in combination with electrospun nanofibrous scaffolds. J Cell Physiol. 2018;233(10):6509‐6522. 29719054 10.1002/jcp.26606

[2] Ng J , Spiller K , Bernhard J , et al. Biomimetic approaches for bone tissue engineering. Tissue Eng Part B Rev. 2017;23(5):480‐493. 27912680 10.1089/ten.teb.2016.0289 PMC5653138

[3] Baptista LS , Kronemberger GS , Cortes I , et al. Adult stem cells spheroids to optimize cell colonization in scaffolds for cartilage and bone tissue engineering. Int J Mol Sci. 2018;19(5):1285. 10.3390/ijms19051285 PMC5983745 29693604

[4] Walmsley GG , Ransom RC , Zielins ER , et al. Stem cells in bone regeneration. Stem Cell Rev Rep. 2016;12(5):524‐529. 27250635 10.1007/s12015-016-9665-5 PMC5053855

[5] McGovern JA , Griffin M , Hutmacher DW . Animal models for bone tissue engineering and modelling disease. Dis Model Mech. 2018;11(4):dmm033084. 29685995 10.1242/dmm.033084 PMC5963860

[6] Travnickova M , Bacakova L . Application of adult mesenchymal stem cells in bone and vascular tissue engineering. Physiol Res. 2018;67(6):831‐850. 30204468 10.33549/physiolres.933820

[7] Kuang G‐M , Yau W , Lu WW , et al. Osteointegration of soft tissue grafts within the bone tunnels in anterior cruciate ligament reconstruction can be enhanced. Knee Surg Sports Traumatol Arthrosc. 2010;18(8):1038‐1051. 19779894 10.1007/s00167-009-0910-1

[8] Nakashima T , Hayashi M , Takayanagi H . New insights into osteoclastogenic signaling mechanisms. Trends Endocrinol Metab. 2012;23(11):582‐590. 22705116 10.1016/j.tem.2012.05.005

[9] Elefteriou F , Benson MD , Sowa H , et al. ATF4 mediation of NF1 functions in osteoblast reveals a nutritional basis for congenital skeletal dysplasiae. Cell Metab. 2006;4(6):441‐451. 17141628 10.1016/j.cmet.2006.10.010 PMC2756713

[10] Chen L , Zou X , Zhang RX , et al. IGF1 potentiates BMP9‐induced osteogenic differentiation in mesenchymal stem cells through the enhancement of BMP/Smad signaling. BMB Rep. 2016;49(2):122‐127. 26645636 10.5483/BMBRep.2016.49.2.228 PMC4915116

[11] Cui Q , Xing J , Yu M , et al. Mmu‐miR‐185 depletion promotes osteogenic differentiation and suppresses bone loss in osteoporosis through the Bgn‐mediated BMP/Smad pathway. Cell Death Dis. 2019;10(3):172. 30787286 10.1038/s41419-019-1428-1 PMC6382812

[12] Yang F , Yang D , Tu J , et al. Strontium enhances osteogenic differentiation of mesenchymal stem cells and in vivo bone formation by activating Wnt/catenin signaling. Stem Cells. 2011;29(6):981‐991. 21563277 10.1002/stem.646

[13] Tian Y , Xu Y , Xue T , et al. Notch activation enhances mesenchymal stem cell sheet osteogenic potential by inhibition of cellular senescence. Cell Death Dis. 2017;8(2):e2595. 28151468 10.1038/cddis.2017.2 PMC5386477

[14] He Y , Zou L . Notch‐1 inhibition reduces proliferation and promotes osteogenic differentiation of bone marrow mesenchymal stem cells. Exp Ther Med. 2019;18(3):1884‐1890. 31410150 10.3892/etm.2019.7765 PMC6676088

[15] Yang S , Guo L , Su Y , et al. Nitric oxide balances osteoblast and adipocyte lineage differentiation via the JNK/MAPK signaling pathway in periodontal ligament stem cells. Stem Cell Res Ther. 2018;9(1):118. 29716662 10.1186/s13287-018-0869-2 PMC5930947

[16] Zheng W , Gu X , Sun X , et al. FAK mediates BMP9‐induced osteogenic differentiation via Wnt and MAPK signaling pathway in synovial mesenchymal stem cells. Artif Cells Nanomed Biotechnol. 2019;47(1):2641‐2649. 31240956 10.1080/21691401.2019.1631838

[17] Yang H , Guo Y , Wang D , et al. Effect of TAK1 on osteogenic differentiation of mesenchymal stem cells by regulating BMP‐2 via Wnt/beta‐catenin and MAPK pathway. Organogenesis. 2018;14(1):36‐45. 29913119 10.1080/15476278.2018.1455010 PMC6150051

[18] Camp E , Anderson PJ , Zannettino ACW , et al. Tyrosine kinase receptor c‐ros‐oncogene 1 mediates TWIST‐1 regulation of human mesenchymal stem cell lineage commitment. Bone. 2017;94:98‐107. 27669657 10.1016/j.bone.2016.09.019

[19] Antonopoulou I , Mavrogiannis LA , Wilkie AO , et al. Alx4 and Msx2 play phenotypically similar and additive roles in skull vault differentiation. J Anat. 2004;204(6):487‐499. 15198690 10.1111/j.0021-8782.2004.00304.x PMC1571319

[20] Thrivikraman G , Boda SK , Basu B . Unraveling the mechanistic effects of electric field stimulation towards directing stem cell fate and function: a tissue engineering perspective. Biomaterials. 2018;150:60‐86. 29032331 10.1016/j.biomaterials.2017.10.003

[21] Burke DP , Khayyeri H , Kelly DJ . Substrate stiffness and oxygen availability as regulators of mesenchymal stem cell differentiation within a mechanically loaded bone chamber. Biomech Model Mechanobiol. 2015;14(1):93‐105. 24832965 10.1007/s10237-014-0591-7

[22] Heydari Asl S , Hosseinpoor H , Parivar K , et al. Physical stimulation and scaffold composition efficiently support osteogenic differentiation of mesenchymal stem cells. Tissue Cell. 2018;50:1‐7. 29429509 10.1016/j.tice.2017.11.001

[23] Rasi Ghaemi S , Delalat B , Ceto X , et al. Synergistic influence of collagen I and BMP 2 drives osteogenic differentiation of mesenchymal stem cells: a cell microarray analysis. Acta Biomater. 2016;34:41‐52. 26196081 10.1016/j.actbio.2015.07.027

[24] Fideles SOM , Ortiz AC , Assis AF , et al. Effect of cell source and osteoblast differentiation on gene expression profiles of mesenchymal stem cells derived from bone marrow or adipose tissue. J Cell Biochem. 2019;120(7):11842‐11852. 10.1002/jcb.28463 30746760

[25] AlMuraikhi N , Ali D , Alshanwani A , et al. Stem cell library screen identified ruxolitinib as regulator of osteoblastic differentiation of human skeletal stem cells. Stem Cell Res Ther. 2018;9(1):319. 30463599 10.1186/s13287-018-1068-x PMC6249887

[26] Anam K , Davis TA . Comparative analysis of gene transcripts for cell signaling receptors in bone marrow‐derived hematopoietic stem/progenitor cell and mesenchymal stromal cell populations. Stem Cell Res Ther. 2013;4(5):112. 24405801 10.1186/scrt323 PMC3854681

[27] Granchi D , Ochoa G , Leonardi E , et al. Gene expression patterns related to osteogenic differentiation of bone marrow‐derived mesenchymal stem cells during ex vivo expansion. Tissue Eng Part C Methods. 2010;16(3):511‐524. 19686055 10.1089/ten.TEC.2009.0405

[28] Hamidouche Z , Fromigue O , Ringe J , et al. Priming integrin alpha5 promotes human mesenchymal stromal cell osteoblast differentiation and osteogenesis. Proc Natl Acad Sci USA. 2009;106(44):18587‐18591. 19843692 10.1073/pnas.0812334106 PMC2773973

[29] Alves RD , Eijken M , van de Peppel J , et al. Calcifying vascular smooth muscle cells and osteoblasts: independent cell types exhibiting extracellular matrix and biomineralization‐related mimicries. BMC Genom. 2014;15:965. 10.1186/1471-2164-15-965 PMC4247655 25380738

[30] Zhou Y , Zhou B , Pache L , et al. Metascape provides a biologist‐oriented resource for the analysis of systems‐level datasets. Nat Commun. 2019;10(1):1523. 30944313 10.1038/s41467-019-09234-6 PMC6447622

[31] Kanehisa M . The KEGG database. Novartis Foundation symposium. 2002;247:91‐101; discussion ‐3, 19‐28, 244‐52. 12539951

[32] Kanehisa M , Furumichi M , Tanabe M , et al. KEGG: new perspectives on genomes, pathways, diseases and drugs. Nucleic Acids Res. 2017;45(D1):D353‐D361. 27899662 10.1093/nar/gkw1092 PMC5210567

[33] Pomaznoy M , Ha B , Peters B . GOnet: a tool for interactive gene ontology analysis. BMC Bioinform. 2018;19(1):470. 10.1186/s12859-018-2533-3 PMC6286514 30526489

[34] Smoot ME , Ono K , Ruscheinski J , et al. Cytoscape 2.8: new features for data integration and network visualization. Bioinformatics. 2011;27(3):431‐432. 21149340 10.1093/bioinformatics/btq675 PMC3031041

[35] Demchak B , Hull T , Reich M , et al. Cytoscape: the network visualization tool for GenomeSpace workflows. F1000Res. 2014;3:151. 25165537 10.12688/f1000research.4492.1 PMC4133763

[36] Petho A , Chen Y , George A . Exosomes in extracellular matrix bone biology. Curr Osteoporos Rep. 2018;16(1):58‐64. 29372401 10.1007/s11914-018-0419-y PMC5812795

[37] Freeman FE , Browe DC , Nulty J , et al. Biofabrication of multiscale bone extracellular matrix scaffolds for bone tissue engineering. Eur Cell Mater. 2019;38:168‐187. 31602629 10.22203/eCM.v038a12

[38] Jeon J , Lee MS , Yang HS . Differentiated osteoblasts derived decellularized extracellular matrix to promote osteogenic differentiation. Biomater Res. 2018;22:4. 29484201 10.1186/s40824-018-0115-0 PMC5824473

[39] Verrier S , Alini M , Alsberg E , et al. Tissue engineering and regenerative approaches to improving the healing of large bone defects. Eur Cell Mater. 2016;32:87‐110. 27434267 10.22203/ecm.v032a06

[40] Smith LR , Cho S , Discher DE . Stem cell differentiation is regulated by extracellular matrix mechanics. Physiology. 2018;33(1):16‐25. 29212889 10.1152/physiol.00026.2017 PMC5866410

[41] Peng WX , Gao CH , Huang GB . High throughput analysis to identify key gene molecules that inhibit adipogenic differentiation and promote osteogenic differentiation of human mesenchymal stem cells. Exp Ther Med. 2019;17(4):3021‐3028. 30936973 10.3892/etm.2019.7287 PMC6434248

[42] Feister HA , Onyia JE , Miles RR , et al. The expression of the nuclear matrix proteins NuMA, topoisomerase II‐alpha, and ‐beta in bone and osseous cell culture: regulation by parathyroid hormone. Bone. 2000;26(3):227‐234. 10709994 10.1016/s8756-3282(99)00269-0

[43] Yamagishi T , Otsuka E , Hagiwara H . Reciprocal control of expression of mRNAs for osteoclast differentiation factor and OPG in osteogenic stromal cells by genistein: evidence for the involvement of topoisomerase II in osteoclastogenesis. Endocrinology. 2001;142(8):3632‐3637. 11459812 10.1210/endo.142.8.8310

[44] Feister HA , Swartz D , Odgren PR , et al. Topoisomerase II expression in osseous tissue. J Cell Biochem. 1997;67(4):451‐465. 9383705

[45] Heo SK , Noh EK , Gwon GD , et al. LIGHT (TNFSF14) increases the survival and proliferation of human bone marrow‐derived mesenchymal stem cells. PLoS One. 2016;11(11):e0166589. 27835685 10.1371/journal.pone.0166589 PMC5106019

[46] Dhoke NR , Kalabathula E , Kaushik K , et al. Histone deacetylases differentially regulate the proliferative phenotype of mouse bone marrow stromal and hematopoietic stem/progenitor cells. Stem Cell Res. 2016;17(1):170‐180. 27394013 10.1016/j.scr.2016.07.001

[47] Petry S . Mechanisms of mitotic spindle assembly. Annu Rev Biochem. 2016;85:659‐683. 27145846 10.1146/annurev-biochem-060815-014528 PMC5016079

[48] Agarwal S , Varma D . How the SAC gets the axe: Integrating kinetochore microtubule attachments with spindle assembly checkpoint signaling. Bioarchitecture. 2015;5(1–2):1‐12. 26430805 10.1080/19490992.2015.1090669 PMC4832446

[49] Tuncay H , Ebnet K . Cell adhesion molecule control of planar spindle orientation. Cell Mol Life Sci. 2016;73(6):1195‐1207. 26698907 10.1007/s00018-015-2116-7 PMC11108431

[50] Hemmat M , Castle BT , Odde DJ . Microtubule dynamics: moving toward a multi‐scale approach. Curr Opin Cell Biol. 2018;50:8‐13. 29351860 10.1016/j.ceb.2017.12.013 PMC5911414

[51] Wu J , Akhmanova A . Microtubule‐organizing centers. Annu Rev Cell Dev Biol. 2017;33:51‐75. 28645217 10.1146/annurev-cellbio-100616-060615

[52] Tolic IM , Novak M , Pavin N . Helical twist and rotational forces in the mitotic spindle. Biomolecules. 2019;9(4):132. 10.3390/biom9040132 PMC6523234 30939864

[53] Srivastava S , Panda D . A centrosomal protein STARD9 promotes microtubule stability and regulates spindle microtubule dynamics. Cell Cycle. 2018;17(16):2052‐2068. 30160609 10.1080/15384101.2018.1513764 PMC6260213

[54] Larsson VJ , Jafferali MH , Vijayaraghavan B , et al. Mitotic spindle assembly and gamma‐tubulin localisation depend on the integral nuclear membrane protein Samp1. J Cell Sci. 2018;131(8):jcs211664. 29514856 10.1242/jcs.211664 PMC5963844

[55] Gadadhar S , Bodakuntla S , Natarajan K , et al. The tubulin code at a glance. J Cell Sci. 2017;130(8):1347‐1353. 28325758 10.1242/jcs.199471

[56] di Pietro F , Echard A , Morin X . Regulation of mitotic spindle orientation: an integrated view. EMBO Rep. 2016;17(8):1106‐1130. 27432284 10.15252/embr.201642292 PMC4967962

[57] Tolic IM . Mitotic spindle: kinetochore fibers hold on tight to interpolar bundles. Eur Biophys J. 2018;47(3):191‐203. 28725997 10.1007/s00249-017-1244-4 PMC5845649

[58] Zwetsloot AJ , Tut G , Straube A . Measuring microtubule dynamics. Essays Biochem. 2018;62(6):725‐735. 30287587 10.1042/EBC20180035 PMC6281472

[59] Roostalu J , Surrey T . Microtubule nucleation: beyond the template. Nat Rev Mol Cell Biol. 2017;18(11):702‐710. 28831203 10.1038/nrm.2017.75

[60] Brouhard GJ , Rice LM . Microtubule dynamics: an interplay of biochemistry and mechanics. Nat Rev Mol Cell Biol. 2018;19(7):451‐463. 29674711 10.1038/s41580-018-0009-y PMC6019280

