# Supplementary figures and images for: Bioinformatics analysis of the biological changes involved in the osteogenic differentiation of human mesenchymal stem cells
Source: J Cell Mol Med. 2020 May 28;24(14):7968–78. doi: 10.1111/jcmm.15429 (PMC7348183; doi:10.1111/jcmm.15429)

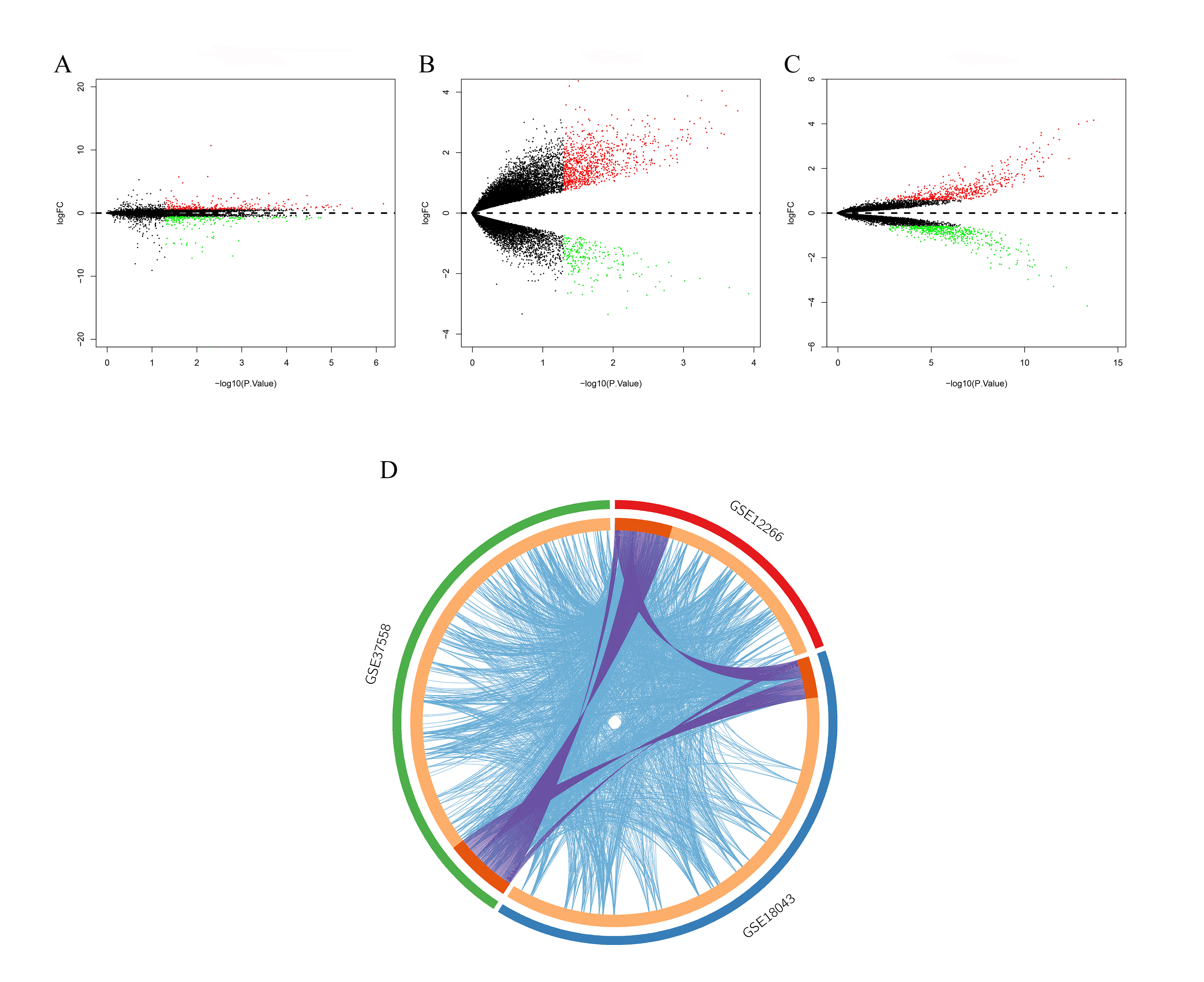

Supplement: Supplementary file 1 — Fig S1 [file JCMM-24-7968-s001.tif]

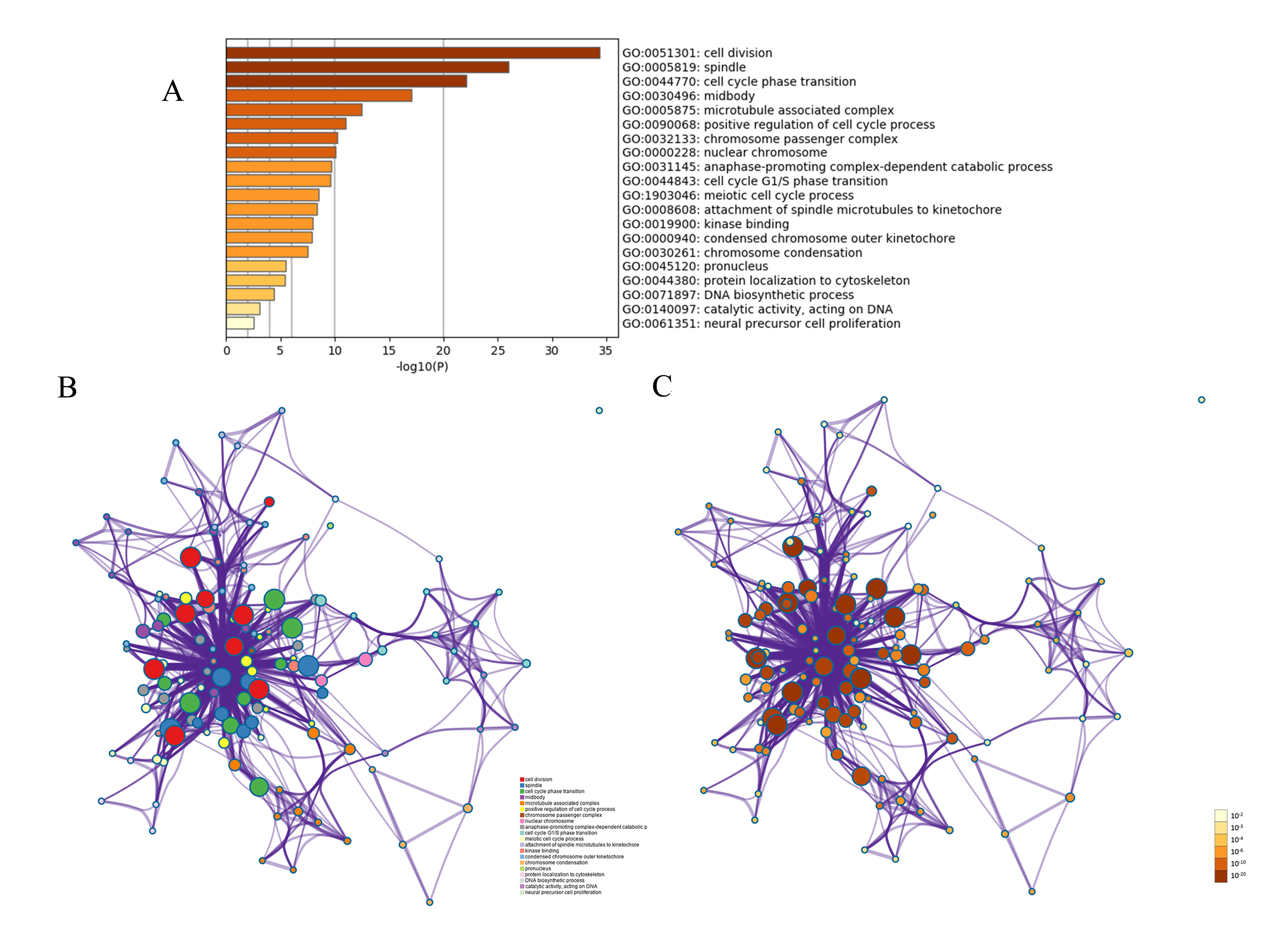

Supplement: Supplementary file 2 — Fig S2 [file JCMM-24-7968-s002.tif]
