# Supplementary material for: Bioinformatics analysis of the biological changes involved in the osteogenic differentiation of human mesenchymal stem cells
Source: J Cell Mol Med. 2020 May 28;24(14):7968–78. doi: 10.1111/jcmm.15429 (PMC7348183; doi:10.1111/jcmm.15429)
Supplement: Supplementary file 3 — Sup info [file JCMM-24-7968-s003.docx]

**Supplemental information**

**Supplementary table 1 Composition of osteogenic induction medium for the three data sets**

|  | GSE12266 | GSE18043 | GSE37558 | applied to hASCs |
| --- | --- | --- | --- | --- |
| Osteogenic differentiation medium | 10 mM β-glycerophosphate, 100 μM ascorbic acid-2 phosphate,  10 nM dexamethasone. | 100 nM dexamethasone. | 5 mM β-glycerophosphate,  0.1 mM Vitamin C,  100 nM dexamethasone. | 100 nM dexamethasone, 37.5 mg/L ascorbic acid, 10 mM-glycerophosphate,  10 nM Vit D3,  2 ng/ml FGF. |

**Figure legends of supplemental information**

**Figure S1 Preliminary comparison of DEGs between the three data sets**

**A-C**: Volcano map of differentially expressed genes (DEGs). The red dot represents the upregulated genes, the green dot represents the downregulated genes, and black dot represents no sense. **A**) GSE2266; **B**) GSE18043; **C**)37558, P<0.05, FC>1.5. **D** An overlapping diagram of three groups of DEGs. Purple curves represent identical genes, blue curves link genes enriched in the same ontology term. The inner circle represents gene lists; genes are arranged along the arc. The dark orange represents the common genes, and light orange represents the unique genes.

**Figure S2 Functional enrichment analysis of genes in the densest region.** (**A**) Bar graph of GO analyses. P value is shown in color. The network of enriched terms of genes; colors represent the same cluster ID (**B**) and p-value (**C**).
